# Drug Release and Cytotoxicity of Hyaluronic Acid and Zinc Oxide Gels, An In-Vitro Study

**DOI:** 10.1080/15685551.2022.2099647

**Published:** 2022-07-24

**Authors:** Jaahnavi Lanka, Santhosh Kumar, Mohana Kumar B, Shama Rao, Shivaprasad Gadag, Usha Y. Nayak

**Affiliations:** a Department of Periodontology, Manipal College of Dental Sciences, Manipal, Manipal Academy of Higher Education, Manipal, Karnataka, India; b Nitte University Centre for Stem Cell Research & Regenerative Medicine, K. S. Hegde Medical Academy, Nitte (Deemed to be University), Mangaluru, Karnataka, India; c Department of Pharmaceutics, Manipal College of Pharmaceutical Sciences, Manipal Academy of Higher Education, Manipal, Karnataka, India

**Keywords:** Dressing, hydrogel, hyaluronic acid, molecular weight, periodontal surgery, wound healing, Zinc-oxide

## Abstract

Hyaluronic acid (HA) is a naturally occurring biopolymer, with a remarkable wound healing property. Zinc-oxide non-eugenol is a material widely used for periodontal dressing in dentistry. However, it has been reported that zinc oxide non-eugenol is toxic to osteoblasts and fibroblasts. Hence, the present study aimed to evaluate the drug release and cytotoxicity of HA and zinc-oxide gels. Hydrogels of HA and zinc oxide were formulated with carbopol as a carrier. *In vitro* drug release was performed by UV spectrophotometry, dialysis, and vial bag methods. Cytotoxicity assessment of HA and zinc-oxide gels was performed in human periodontal ligament fibroblasts (HPdLF) and human gingival fibroblasts (hGFs). An inverted phase-contrast microscope was used to assess the morphological changes. At 24 and 48 hr, HPdLF cells showed the highest viability in 0.1% low molecular weight-HA (LMW-HA) with a median value of 131.9, and hGFs showed the highest viability in 5% LMW-HA with a median of 129.56. The highest viability of HPdLF cells was observed in 5% high molecular weight-HA (HMW-HA), with a median value of 127.11. hGFs showed the highest viability in 1% HMW-HA with a median value of 97.99. Within the limitations of the present study, we concluded that LMW-HA is more efficient than HMW-HA. Both HPdLF and hGF cells showed complete cell morbidity with zinc-oxide hydrogels. Therefore, zinc oxide-based gels in concentrations as low as 9% could be toxic intraorally to soft tissues that harbor gingival and periodontal ligament fibroblasts.

## Introduction

Bacterial infections, functional and physical forces in the oral cavity during periodontal surgery may affect healing [1]. Thus, in periodontal surgical procedures, ranging from minor, aesthetic surgeries, mucogingival corrections, and flap surgeries, protection of the wound area using a periodontal dressing material came into existence [2]. However, although used initially, the eugenol-based dressings are no longer employed due to their allergic reactions.

Hyaluronic acid (HA) is an innately occurring high molecular weight polysaccharide formed in the human body as a part of a natural wound healing mechanism. HA acts as an essential element of cell migration and proliferation, thus regulating tissue hydrodynamics [3]. In addition, it has ideal properties, such as non-immunogenicity and biocompatibility with human oral tissues. The essential cellular interactions of HA, with various mediators, including CD44, a receptor for HA-mediated motility (RHAMM), and intercellular adhesion molecule-1 (ICAM-1), make HA crucial during each stage of wound healing [4]. Moreover, the adjunctive use of HA demonstrated a significant improvement in the periodontal clinical parameters [5–8]. Recently, multi-dimensional studies conducted on HA-based biomembranes [9], mouthwashes [10], local drug delivery [11], as a diagnostic marker of inflammation [12], in bone regeneration [13], and as an adjunct in the management of peri-implantitis [14], can be attributed to its versatility.

Zinc oxide non-eugenol is a widely used material for periodontal wound dressings in dentistry. The antibacterial reaction from metal oxide and fatty acids acts as a barrier to protect wounds [15,16]. Topical zinc oxide was well known for its protective, astringent, and antiseptic properties [15,16]. In addition, research on the use of zinc oxide incorporated in gauze demonstrated enhanced healing of leg ulcers in humans and acute wounds in experimental pigs [17]. Due to these beneficial properties, zinc oxide-based periodontal dressings have been widely used in phase-II therapy of periodontal management [18].

Despite the usage, several *in vitro* studies have reported the high toxicity of zinc-oxide non-eugenol to osteoblasts and fibroblasts [19]. Studies revealed that the presence of rosin in zinc oxide non-eugenol dressings can trigger increased inflammatory reactions involving polymorphonuclear neutrophilic leukocytes [20,21]. Furthermore, it was reported that zinc oxide-based dressings could cause inflammation in the applied area, thereby becoming inhibitory to the wound healing process up to 7 days following its application [21]. Hence, the need for research on newer and safe agents, such as HA as a substitute for zinc oxide-based periodontal surgical dressings, is of high clinical significance.

HA is available in two forms [22]; a low molecular weight HA (LMW-HA) shows angiogenesis, whereas high molecular weight (HMW-HA) has an opposite action. The LMW-HA plays a role in signaling tissue damage and mobilizing immune cells. In contrast, the HMW-HA suppresses the immune response preventing excessive exacerbations of inflammation [4,23]. The differences in viscoelasticity were found to be the reason for antagonistic actions of LMW-HA and HMW-HA [24].

Unfortunately, the number of studies on wound healing benefits of HA are very scarce, and it is mostly used in either a gel or a liquid form. The most common concentrations used are 0.2, 0.8, and 1%, and they were also commercially formulated from HMW-HA. Studies that used LMW-HA variants are also limited. Gingigel, Hyadent, Hyalugel, Aminogam, and Klirich are a few commercially available products that are sparsely available and are of different concentrations formulated with HMW-HA. As a clinician, choosing these commercially available forms of HMW and LMW-HA for periodontal therapy needs more evidence. Therefore, the present study aimed to evaluate the *in vitro* drug release of HMW-HA and LMW -HA and compare their cytotoxicity with zinc oxide gels. The cytotoxicity assessment was performed using human periodontal ligament and gingival fibroblasts.

## Materials and methods

**Figure 1.**
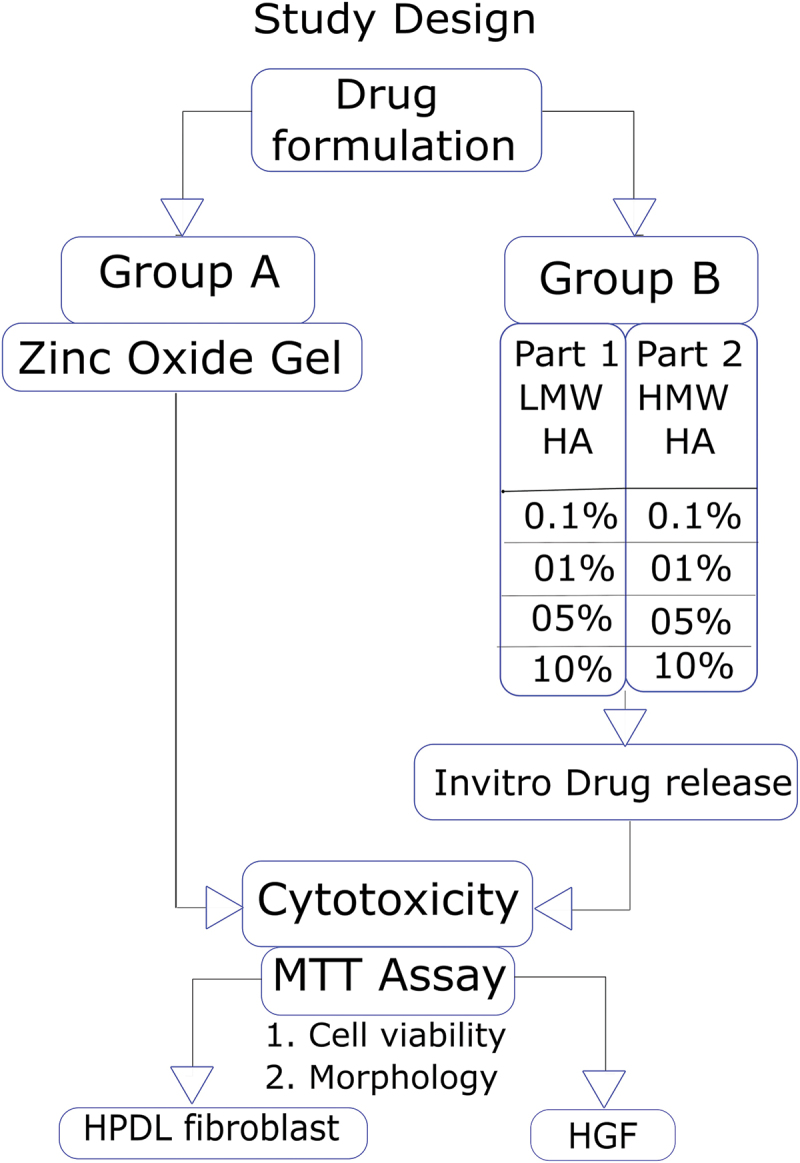
Study design.

The present study is a two-way factorial design. The study protocol is as per CRIS guidelines for *in vitro* studies [25]. The study design is illustrated in Figure 1. The study groups are described in Table 1.

**Table 1.** Study groups.

Group A – Zinc Oxide Gels	Group B – Hyaluronic acid Gels
I. 87% w/v (POSITIVE CONTROL) II. 36% w/v zinc Oxide gel. III. 18% w/v zinc Oxide gel. IV. 9% w/v zinc oxide gel.	**Part 1 – HMW-HA**
	0.1% 1% 5% 10%
	**Part 2 – LMW-HA**
	0.1% 1% 5% 10%

(LMW-HA: Low molecular weight hyaluronic acid; HMW-HA: High molecular weight hyaluronic acid.)

### Drug formulation

#### Preparation of gel base

Gel base was prepared using carbopol 940 (manufactured by Sigma-Aldrich) by soaking in water overnight. 0.1% w/v concentration of carbopol gel was prepared with suitable dilution in water.

#### Hyaluronic acid (HA) gel

HA gel was prepared by dissolving an appropriate amount of HA in 0.1% w/v carbopol gel. The pH of the gel was adjusted to 7.0 using triethanolamine.

#### Zinc oxide gel

Zinc oxide gel was prepared by dissolving an appropriate amount of zinc oxide in 0.1% w/v carbopol gel.

### HMW-HA release study

#### Vial method

Gel was placed in a vial, and pH 7.4 phosphate buffer saline was added. The mixture was kept in a shaking incubator at 37 ± 2°C with 100 rpm. At a predetermined time interval, the sample was collected and centrifuged. We observed that HMW-HA settled down along with the Carbopol due to its high molecular weight, and no HA was present in the supernatant. So, we were not able to perform the release study for the prepared gels using HMW-HA.

## LMW-HA release study

### Dialysis bag method

The molecular weight of HA used was 8 kDa. The cut-off of the dialysis bag was 12 kDa. The sample was collected and centrifuged in a shaking incubator at 37 ± 2°C with 100 rpm at a predetermined time interval.

### Estimation of LMW-HA by UV spectrophotometry

Different concentrations of LMW-HA were prepared in water and analyzed using a UV spectrophotometer at a wavelength of 205 nm. The calibration curve was plotted as a function of absorbance Vs. LMW-HA concentration. The samples of HA and zinc oxide hydrogels were sterilized by gamma irradiation (Gamma Chamber 5000 of Cobalt 60 source) at a dose rate of 2.68 kGy/hr.

### Cytotoxicity assay

The human gingival fibroblasts (hGFs) and periodontal ligament cells (HPdLF) were cultured after procuring tissue fragments from the extracted tooth by the explant technique as described earlier in a study by Kwon et al. [18]. The cultured cells were stored in a MEM-alpha medium (HiMedia) and kept in a CO_2_ incubator. The dead, floating cells were removed by changing the media every two days. Then, the hGFs and HPdLF cells were exposed to the test and control agents. The morphological changes were observed using an inverted phase-contrast microscope and compared at 24, 48, and 72 hr. MTT assay was performed to evaluate the cytotoxicity, and it was matched between the following groups.

### Statistical analysis

SPSS-22 software was used for statistical analysis. The Kruskal–Wallis test was used to analyze the effective concentration in the HA group. In addition, a comparison between all the different concentrations of HA for the viability of cells was performed for both hGFs and HPdLF at three different time points, i.e., 24 hr, 48 hr, and 72 hr by using the Friedman test.

## Results

### In vitro *drug release study*

The drug release study was performed for LMW-HA using the dialysis bag method. The amount of drug released for 0.1% LMW-HA was only 4 hr. After 4 hr timeline, no release was noted. All the other three concentrations of 1%, 5%, and 10% showed drug release for 72 hr, and an increasing trend with time was noticed. The evaluation of *in vitro* release study results showed that 0.1% LMW-HA gel was released only at 1, 2, 3, and 4 hr, and there was no release of HA after 4 hr for 0.1% LMW-HA.

**Figure 2.**
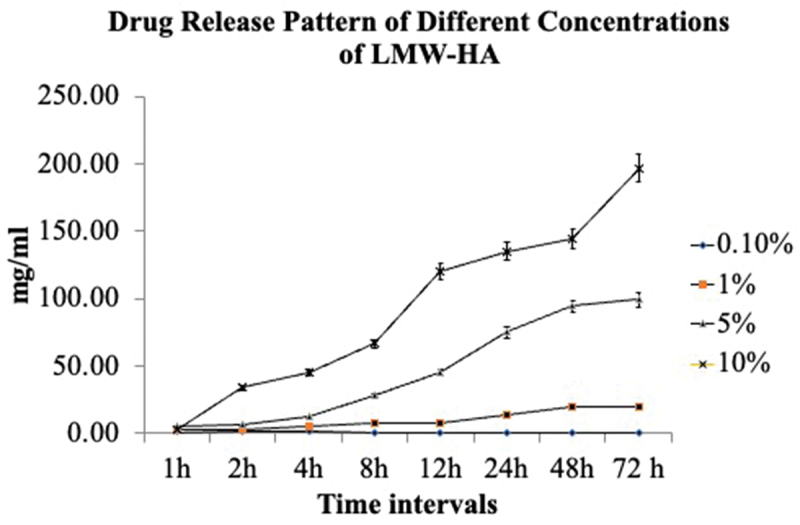
Drug release pattern of different concentrations of LMW-HA by UV spectrophotometry. (LMW-HA: Low molecular weight hyaluronic acid; UV: Ultraviolet).

The other three concentrations released HA gel for up to 72 hr. There was an increase in drug release as the HA concentration increased from 1% to 10%. The release was recorded for up to 72 hr. Drug release patterns are illustrated in Figure 2.

## Cell viability

### Group A – zinc oxide gel

All the four concentrations of zinc oxide gel used, i.e., 87%, 36%, 18%, and 9% (w/v), showed total cell mortality in both hGFs and HPdLF cells.

### Group B – HA gel (Part 1 & 2)

At 24 and 48 hr, the LMW-HA group for HPdLF cells showed the highest viability at 0.1% with a median value of 131.9, and for hGFs, it was 5% with a median of 129.56. For the HMW-HA group, the highest viability for HPdLF cells with a median value of 127.11 was noted in 5% HMW-HA, and for hGFs cells, it was pointed out in 1%, as they showed the highest viability with a median value of 97.99. The HMW-HA group of HPdLF cells and hGFs showed statistically significant results (p < 0005). At 72 hr, it was observed that LMW-HA for both HPdLF and hGFs, and HMW-HA HPdLF cells showed statistically significant results (p < 0.005). A detailed cell viability analysis of HMW-HA and LMW-HA groups at 24, 48 and 72 hours is given in Table 4.

The analysis of the viability of HPdLF cells and hGFs at 24 hr indicated that the highest percentage of viable HPdLF cells were observed in 0.1% LMW-HA and 5% HMW-HA. In the case of hGFs, the highest percentage of viable cells were found in 5% LMW-HA and 1% HMW-HA. The lowest percentage of viable cells in all the groups was 10%, suggesting it was ineffective for all the cells in both LMW and HMW variants (Table 2). The HMW-HA group of HPdLF cells and hGFs showed statistically significant results (p < 0005). The same pattern was noticed at 48 hr (Table 2).

**Table 2.** Comparison of viability values at different time intervals among different concentrations within the groups.

		24 hr			48 hr			72 hr		
	Conc (%)	Median (Min – Max)	IQR	p value	Median (Min – Max	IQR	p value	Median (Min – Max	IQR	P value
LMW-HA-HPDL	0.10	131.93(89.2 − 239.2)	130.5	0.8	103.41 (71.22–198.68)	99.44	0.4	134.68 (130.67 142.59)	9.68	0.008*
	1	125.47 (68.2–141.9)	57.65		95.04 (73.08–106.18)	25.96		67.83 (40.54–86.17)	38.01	
	5	116.74 (78.36–168.49)	78.86		112.74(91.22–119.39) 93.65(78.45–108.39	23.45		95.65 (78.75–107.21)	25.54	
	10	107.65 (87.45–117.24)	24.72			28.16		93.5 (80.82–95.19)	11.05	
LMW-HA-HGF	0.10	97.76 (56.95–162.17)	93.48	0.6	70.69 (51.3–81.95)	23.61	0.9	95.95 (57.86–115.95)	43.65	0.54
	1	106.08 (60.89–150.93)	72.85		62.32 (57.77–104.8)	36.83		91.1 (57.61–112.93)	42.73	
	5	129.56 (52.59–158.23)	91.73		73.68 (52.21–115.14)	49.92		107.38 (72.18–134.43)	46.82	
	10	82.34 (55.0–99.2)	36.59		70.92 (49.83–85.09)	26.65		107.91 (87.81–130.76)	35.26	
HMW-HA-HPDL	0.10	99.15 (81.06–111.33)	24.82	0.02*	99.15 (81.06–111.33)	24.82	0.02*	119.22 (104.51–141.96)	30.14	0.01*
	1	109.39 (93.84–160.59)	51.49		109.39 (93.84–160.59)	51.49		77.22 (62.32–127.31)	50.77	
	5	127.11 (63.73–213.9)	114.38		127.11 (63.73–213.9)	114.38		88.61 (84.05–91.56)	6.05	
	10	50.52 (40.32–59.91)	15.41		50.52 (40.32–59.91)	15.41		59.87 (50.68–61.94)	8.82	
HMW-HA-HGF	0.10	87.62 (46.8–132.9)	70.89	0.03*	87.62 (46.8–132.9)	70.89	0.03*	105.76 (75–136.73)	59.08	0.25
	1	97.99 (89.39–104.17)	11.41		97.99 (89.39–104.17)	11.41		80.71 (71.69–87.79)	13.3	
	5	91.68 (57.78–106.6)	39.65		91.68 (57.78–106.6)	39.65		78.58 (67.51–84.66)	15.64	
	10	22.36 (17.9–33.31)	11.83		22.36 (17.92–33.31)	11.83		65.21 (57.23–91.78)	26.87	

P value calculated using Kruskal–Wallis test; *Significant (LMW-HA: Low molecular weight hyaluronic acid; HMW-HA: High molecular weight hyaluronic acid; HPdLF: Human periodontal ligament fibroblasts; hGFs: Human gingival fibroblasts)

Conversely, at 72 hr, it was noticed that HMW-HA had slightly varied results compared to the other two timelines. The highest percentage of viable HPdLF and hGFs were found in 0.1% HMW-HA. LMW-HA showed similar results at 24- and 48-hr timelines, i.e., for HPdLF cells, 0.1% was found to be effective and 5% for hGFs cells. The results of HPdLF and hGFs cells of LMW-HA and HPdLF cells of HMW-HA groups showed statistically significant results (p < 0.005). (Table 2)

The comparison of viability values within the groups at subsequent time intervals by the Friedman test showed statistically significant changes for HPdLF cells in 0.1% HMW-HA, which showed the highest viability percentage at all the timelines. 10% HMW-HA and LMW-HA showed the statistically significant (p < 0.005) least viability percentage for all the groups except HPdLF cells in 0.1% LMW-HA. (Table 3)

**Table 3.** Comparison of the viability values within the groups at subsequent time intervals.

Variables		0.10%	1.00%	5%	10%
LMW-HA	PDL	0.77	0.17	0.17	0.47
	HGF	0.36	0.36	0.36	0.039*
HMW-HA	PDL	0.039*	0.17	0.17	0.039*
	HGF	0.47	0.36	0.36	0.018*

P value calculated using Friedman test; *Significant (LMW-HA: Low molecular weight hyaluronic acid; HMW-HA: High molecular weight hyaluronic acid; HPdLF: Human periodontal ligament fibroblasts; hGFs: Human gingival fibroblasts)

**Table 4.** Comparison of cell viability between the LMW-HA and HMW-HA groups.

Time intervals	Concentrations	Chi-Square value	p value
24 hr	0.10%	1.985	0.575
	1%	1.963	0.580
	5%	1.875	0.599
	10%	13.125	0.004*
48 hr	0.10%	7.743	0.052
	1%	1.875	0.599
	5%	6.684	0.083
	10%	10.891	0.012*
72 hr	0.10%	6.296	0.098
	1%	2.713	0.438
	5%	5.382	0.146
	10%	11.272	0.010*

*Significant

(LMW-HA: Low molecular weight Hyaluronic acid; HMW-HA: High molecular weight Hyaluronic acid; HPdLF: Human periodontal ligament fibroblasts; hGFs: Human gingival fibroblasts)

A comparison of viability among the groups using the Kruskal–Wallis test indicated that 10% of LMW-HA and HMW-HA had shown (statistically significant) almost negligible percentage of viable HPdLF cells and hGFs at all the three timelines of 24, 48, and 72 hr (Table 3).

### Morphological changes

**Figure 3.**
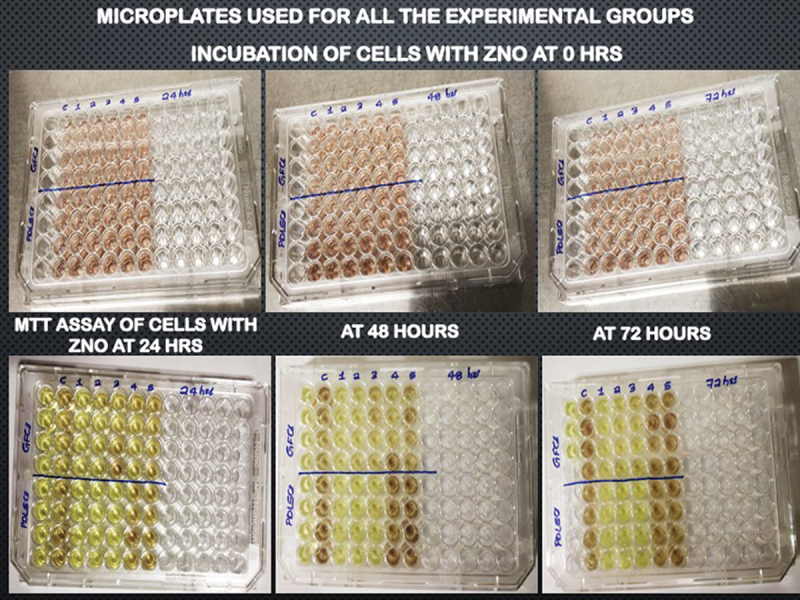
96-Well microplates used for cytotoxicity with all the experimental groups.

During the cytotoxicity assay, any changes in the morphology of HPdLF cells and hGFs were observed using an inverted phase-contrast microscope. The images of 96-well microplates used for the assay are presented in Figure 3. The viable HPdLF and hGFs were observed as elongated, spindle-shaped cells, with fibroblastic morphology displaying proper plastic adherence characteristics. Notably, the zinc oxide group showed completely dead cells, which were observed as round or oval shaped and found floating as suspension in the media.

## Discussion

The investigations that focused on drug release patterns of HMW and LMW-HA *in vitro* are limited. The present study is one of the few to evaluate the *in vitro* release of LMW-HA. Quantification of HMW-HA was difficult due to its high molecular weight and complex molecular structure [26]. In the present study, quantification of HMW-HA posed similar difficulties. Observations from the cell-based study revealed exciting findings regarding the selective action and efficacy of LMW-HA and HMW-HA on HPdLF and hGF cells.

In Group A, 87%, 36%, 18%, and 9% (w/v) of the samples used were zinc oxide-based hydrogels. We had chosen 87% and 36% (w/v) concentrations from the existing evidence of commercially available zinc oxide-based periodontal packs [27]. The other two concentrations were selected by reducing the least available concentration to half, making it 18% and 9%, respectively. It was noticed that there was a complete cell death in both HPdLF cells and hGFs. Hence, we diluted the sample in a 1:100 ratio as described previously, resulting in total cell mortality [28]. These results strongly correlate with the present study as many other studies on the toxicity of zinc oxide on human cell lines, questioning the use of zinc oxide in commercially available periodontal dressings used on periodontal surgical wounds [29,30].

At the 24-hr timeline, for HPdLF cells, LMW-HA showed viabilities in the following order: 0.1%>1%, then, 5%>10%, suggesting 0.1% to be more effective and 10% to be least effective. This finding indicates that 0.1% has a physical property, suitable for HPdLF cells, which might have favored the proliferation of cells. On the other hand, HMW-HA gels showed viabilities differently, i.e., 5%>1%>0.1%>10%. These trends in viability indicate that the most effective concentration is 5% HMW-HA for HPdLF cells. This could be because of the mucoadhesive property of high molecular weight, which can stay longer with the HPdLF cells.^26^

hGFs exhibited the viability to LMW-HA in the order of 5%>1%>0.1%>10% and to HMW-HA in the order of 1%>5%>0.1%>10%. Hence, the effective concentrations of LMW-HA and HMW-HA for hGFs were 5% LMW-HA and 1% HMW-HA, respectively. It can be inferred that the liquid-type consistency of 5% LMW-HA and 1% HMW-HA is favourable for the survival and proliferation of hGFs, unlike the HPdLF cells. In all the groups, 10% concentration was observed to be ineffective with the lowest viability percentage. This ineffectiveness of 10% HA gel can be attributed to the rheological properties of HA. This was in correlation with previous studies on the rheological properties of HA [26,31–36]. The inferences of viability at 24 hr are similar as observed at 48 hr of the study, with minor differences at 72 hr.

The effectiveness of LMW-HA over HMW-HA could be related to its immunoregulatory property, which is usually noticed at the sites of inflammation. LMW-HA is formed from enzymatic degradation of HMW-HA. LMW-HA promotes the production of immune mediators thereby stimulating an immune response. This could enhance wound healing better than the high molecular weight variant [37]. Hence, it is observed that LMW-HA showed better viability percentages than HMW-HA in both HPdLF cells and hGFs, which needs to be confirmed in further large-scale randomized clinical trials.

A similar pattern of gamma irradiation was performed to prepare LMW-HA and found that it showed statistically significant benefits of wound healing *in vitro* on human skin cell lines [32].

In another randomized placebo-controlled clinical trial, there were no statistically significant benefits of HA-applied sites after surgery compared to placebo sites [36]. This may be because the effective form that could induce the proliferation of cells was not used. A randomized controlled trial studied the efficacy of HA wound dressings and compared them to povidone-iodine. Wound healing assessments were conducted using the Bates Jensen wound assessment tool. They have concluded that HA-based wound dressings accelerated the rate of granulation tissue formation and thereby wound healing as compared to povidone-iodine [38]. In a recent study conducted in diabetic rats, an injectable multifunctional hydrogel based on dopamine-grafted HA and phenyl boric acid-grafted methylcellulose was fabricated for promoting the repair of diabetic wounds. It was concluded that HA-based hydrogels possessed great potential in chronic wound dressings [39]. Another study in a large skin wound model used a biofunctional hydrogel in which mesenchymal stem cell-derived small extracellular vesicles were incorporated into the injectable hyaluronic acid hydrogel and found that they induced immunomodulatory effects, thereby leading to a scarless wound healing [40].

## Conclusion

After a comprehensive analysis of the results, we could arrive at a conclusion that

1. The effective concentration of HMW-HA for hGFs is found to be 1%. Above 1% concentration is inhibitory to hGF cells.
2. The effective concentration of HMW-HA for HPdLF cells is found to be 5%.
3. The effective concentration of LMW-HA for hGFs is found to be 5%.
4. The effective concentration of LMW-HA for HPdLF cells is found to be 0.1%
5. LMW-HA is found to be more effective than HMW-HA in both HPdLF cells and hGFs.
6. The mortality of both HPdLF cells and hGFs in vitro to concentrations even as low as 9% of zinc oxide- based gels have shown to be very toxic. Hence, exposing this material intraorally to soft tissues that harbor gingival and periodontal ligament fibroblasts is questionable.
